# Investigating the Social Network Structure of Physical Literacy Scholars to Advance a Paradigm for Physical Activity Promotion

**DOI:** 10.3389/fspor.2022.809946

**Published:** 2022-04-12

**Authors:** Leeann M. Lower-Hoppe, Amy Chan Hyung Kim, Shea M. Brgoch, Christin M. Zwolski, Laura Schmitt, Matthew K. Paponetti, Catherine C. Quatman-Yates

**Affiliations:** ^1^Department of Human Sciences, The Ohio State University, Columbus, OH, United States; ^2^Department of Sport Management, Florida State University, Tallahassee, FL, United States; ^3^School of Kinesiology, Recreation & Sport, Western Kentucky University, Bowling Green, KY, United States; ^4^Division of Occupational Therapy and Physical Therapy, Cincinnati Children's Hospital, Cincinnati, OH, United States; ^5^School of Health and Rehabilitation Sciences, The Ohio State University, Columbus, OH, United States; ^6^Nationwide Children's Hospital, Sports and Orthopedic Physical Therapy, Columbus, OH, United States

**Keywords:** sociology of scientific knowledge, social network analysis, co-authorship analysis, scientific collaborations, underserved populations

## Abstract

**Purpose:**

Physical literacy has quickly gained global attention as a holistic approach to combat physical inactivity and obesity. However, research silos may limit the growth and application of the physical literacy paradigm for effective physical activity promotion. The purpose of this study was to measure the underlying network structure of scholars publishing on physical literacy (focusing on empirical research) through co-authorship analysis.

**Methods:**

Data collection resulted in 1,070 documents related to physical literacy retrieved. A total of 198 articles met inclusion criteria and were included in the full network, with authors operationalized as actors in the network. A total of 75 empirical studies were included in the sub-network for critical appraisal and further analysis. Social network analysis was then conducted at the macro- and component-level, using quantification and visualization techniques.

**Results:**

Results revealed a collaborative, yet fragmented physical literacy network with sub-groups representing substantive and geographically diverse scholars. The majority of scholarship lacked empirical evidence, suggesting a research-practice gap.

**Conclusion:**

Recommendations for advancing physical literacy research and practice include strategic collaborations that transcend geographic and disciplinary boundaries, cooperative efforts across scholars and practitioners, and productive discourse through professional avenues to progress knowledge generation, dispersion, and application.

## Introduction

Physical inactivity is considered one of the most significant public health issues of the twenty-first century based on its prevalence, global reach, and health effect (Blair, 2009; Kohl et al., 2012). The World Health Organization (WHO) estimates 81% of adolescents (age 11–17 years) and 27.5% of adults (age 18 years and older) do not meet physical activity recommendations worldwide (Guthold et al., 2018, 2020). Insufficient physical activity can lead to secondary health conditions (e.g., obesity), diminished quality of life, and reduced life expectancy (World Health Organization, 2020). Across the globe, obesity rates have nearly tripled since 1975, with increasing rates of physical inactivity identified as a primary cause of obesity (World Health Organization, 2020). Underserved populations, including individuals with disabilities, low socioeconomic status (SES), and minority racial/ethnic backgrounds (among others), are particularly at risk for sedentary behaviors, obesity, and secondary health conditions due to physical, social, and economic disadvantages (Mendoza-Vasconez et al., 2016; Bopp et al., 2019).

From an ecological perspective, individual (e.g., ability, needs, motivation), interpersonal (e.g., social support, cultural norms), and environmental (e.g., safety, access to facilities) factors often impede physical activity engagement among underserved populations (Bauman et al., 2012). For example, individuals with intellectual disabilities experience complex social, physical, and cognitive challenges; reduced educational and employment opportunities; and increased rates of poverty—compared to their typically developing peers—that impact access and opportunity to engage in a physically active lifestyle (Abidi and Sharma, 2014). Cumulative sociocultural, educational, health, and economic disadvantages impacting underserved populations increase their exposure to health risk factors and perpetuate health disparities (Bopp et al., 2019). To break the cycle of health disparities, scholars and practitioners alike have advocated for the promotion of physical literacy (The Aspen Institute, 2015).

*Physical literacy* has re-emerged as a holistic approach to combat physical inactivity and obesity among diverse populations (Cairney et al., 2019b). While physical literacy has gained global attention, there is ongoing debate around the construct, with scholars seeking to clarify the definitions and foundations of physical literacy (see Edwards et al., 2017; Shearer et al., 2018; Cairney et al., 2019b). Broadly, physical literacy encompasses affective (e.g., confidence, motivation), cognitive (e.g., knowledge, understanding), and physical (e.g., fundamental movement skills, physical competence) properties that influence one's engagement in physical activity across the lifespan (Edwards et al., 2017; Shearer et al., 2018; Martins et al., 2021). Individuals (children and adults) progress through a unique lifelong physical literacy journal, interacting with the environment around them to learn and develop through movement. The philosophical underpinnings of physical literacy draw from a monist ontology (i.e., a person's mind and body work together) and phenomenological (i.e., a person perceives the world from their unique point of view) and existential (i.e., a person's existence precedes their essence) epistemology (Edwards et al., 2017).

The adoption of physical literacy as a holistic approach to optimize the human movement experience is found in a subset of countries (e.g., United Kingdom, Canada, Australia, United States, Ireland; Mitchell and Le Masurier, 2014; Edwards et al., 2017) and is increasingly reaching journals tied to a variety of movement-related disciplines such as physical education, recreation and leisure, sport management, and sport medicine (Mitchell and Le Masurier, 2014; Dudley et al., 2017; Edwards et al., 2017; Zwolski et al., 2017; Roetert et al., 2018; Cairney et al., 2019a). Physical literacy has attracted scholars' attention due to its potential to promote healthy development of the whole person, especially when foundations are built during early childhood (Edwards et al., 2017; Dania et al., 2020). An outcome of the widespread interest in physical literacy across diverse countries and academic disciplines is an expansion of the community of scholars engaged in the physical literacy idea space.

Physical literacy theorists have recognized “independent research groups currently operationalize the construct differently” (Edwards et al., 2017, p. 113), leading to confusion over the properties, philosophical foundations, and measurement of physical literacy (Longmuir and Tremblay, 2016). For example, physical literacy has varied representations across the world (Shearer et al., 2018; Martins et al., 2021), such as the Canadian multi-institutional model, SHAPE America single-institutional model, and Aspen Institute promotional model (Corbin, 2016). Physical literacy can also be framed to represent an individual's physical interactivity with others and the world to enrich one's overall life experience (i.e., corporeal intra/interactivity), or framed as becoming physically active in order to reach a state of fitness and health (i.e., physical activity habitus; Babcock, 2020, 2021). When measuring physical literacy, scholars have either employed holistic tools to capture the multidimensionality of physical literacy (e.g., Canadian Assessment of Physical Literacy; Francis et al., 2016) or a compilation of tools focusing on individual dimensions (e.g., Test of Gross Motor Development-3; Houser et al., 2019). The varied philosophies, frameworks, and measurements can contribute to confusion within the physical literacy idea space, which may limit the growth and application of a coherent paradigm (model that guides coherent traditions of scientific practice and research; Kuhn, 1970).

Scientific revolution theorists assert scientific knowledge of a paradigm grows systematically as scientists examine the fundamental problems outlined by the paradigm and build upon prior scholars' work (Kuhn, 1970; Crane, 1988). The study of science as a social system treats scientific knowledge not as a body of literature, but rather the organized activity of scholars concerned with extending knowledge through use of scientific techniques (Storer, 1966). The process of scientists building a cumulative and coherent body of knowledge involves scientists being socially influenced by and socially influencing other scientists, illustrating a “contagion” effect within a community of scholars (Crane, 1988; Locher, 2002).

The sociology of scientific knowledge indicates the success of a given paradigm is largely determined by the social interactions among scholars working in that idea space (Moody, 2004; Quatman and Chelladurai, 2008b; Johnson et al., 2016). The pattern of social interactions among scholars influences the content, consensus, and application of the paradigm (Crane, 1972; Moody, 2004; Johnson et al., 2016). Direct and indirect ties within a social network of scientists influence the diffusion of ideas, resources, and information, which can lead to knowledge generation, cross-fertilization, and subject dispersion, further extending the development, understanding, and application of a paradigm (Crane, 1988; Fonseca et al., 2016).

While diversity of perspectives is important to broaden and improve a paradigm for application across fields, cooperative efforts to promote progression of a cohesive paradigm are needed to advance the potential impact of putting the scientific paradigm into practice (Crane, 1972, 1988; Moody, 2004; Quatman and Chelladurai, 2008b). Moreover, a well-connected physical literacy scientific community can produce a common language around a unified paradigm to progress knowledge generation, dispersion, and application for effective physical activity promotion. Comparatively, a physical literacy scientific community lacking cohesion may produce competing frameworks that inhibit or slow down the advancement of physical literacy strategies to address physical inactivity and obesity.

To promote the growth of the physical literacy paradigm for effective physical activity promotion, the current status and structure of the physical literacy scientific community must be revisited. For this, social network analysis (SNA) can be employed to characterize and quantify the social structure and interaction among scholars contributing to the physical literacy idea space (Freeman, 2004; Quatman and Chelladurai, 2008b). Broadly, a network refers to the web or pattern of relationships (ties) within a social system (Nadel, 1957). As the “total network” of the social world is constantly reticulating, stretching past the boundaries of any community, research must focus on particular aspects of the total network (Mitchell, 1969; Scott, 2000). An “ego-centered network” is anchored around the social relations of singular individuals, while a “global network” examines relational webs around a particular social phenomenon (Scott, 2000). The global network of the physical literacy scientific community is the focus of the current study.

Scholars within the physical literacy scientific community have identified an absence of empirical research to support the operationalizing, theorizing, and measuring of physical literacy (Edwards et al., 2017; Hyndman and Pill, 2018; Shearer et al., 2018). Moreover, Edwards et al. (2018) articulated a need for “undertaking empirical research, to ensure alignment between the definition, philosophy, and measure/assessment [of physical literacy] adopted” (p. 679). Empirical research can help translate physical literacy from “an abstract theoretical concept into a tractable measurable entity” (Edwards et al., 2018, p. 661), to enable the development of evidence-based physical literacy interventions that promote physical activity across the lifespan. In light of this gap in the literature, the authors of the current study centered their examination of the global network of the physical literacy scientific community on scholars engaged in empirical research.

One of the most visible indicators of social interactions advancing a scientific paradigm is co-authorship on publications. Co-authorship analysis using SNA techniques is an established method to measure the underlying network structure of a community of scholars (Moody, 2004; Hazlett et al., 2005; Quatman and Chelladurai, 2008a). Co-authorship analysis has the potential to identify leading collaborations that could serve as scientific bridges in the scientific community, assess opportunities for interdisciplinary collaboration, illuminate areas of (mis)alignment for advancement of a unified paradigm, and inform strategic innovations to address complex problems (Fonseca et al., 2016). To date, only one scientometric analysis of physical literacy authors has been conducted (see Young et al., 2021), with a focus on the most published physical literacy authors (i.e., authored more than five physical literacy articles). The current study expands upon this work by investigating the social network of all authors in the physical literacy idea space, with a focus on authors engaged in physical literacy empirical scholarship.

Considering the diversity of the physical literacy scientific community, there is potential for scholars engaged in defining and testing models of physical literacy to be working in relative isolation and producing scholarship independent or in contention with one another. There is opportunity to clarify and advance the physical literacy paradigm through an examination of the social network structure among scholars publishing on physical literacy. With an understanding of the primary actors informing the physical literacy paradigm and their associated works, the scientific community can reach a level of transparency necessary for a coherent paradigm that enables physical literacy to be globally operationalized and leveraged for physical activity promotion. The purpose of this study is to investigate the social network structure of the community of physical literacy scholars to refine and extend our understanding and application of the physical literacy paradigm as a holistic approach to promote physical activity. The following research questions guided our investigation:

RQ1: What is the social network structure of the global network of authors publishing on physical literacy (i.e., full network)?
RQ2: What is the social network structure of the global sub-network of authors publishing empirical research on physical literacy (i.e., empirical sub-network).

## Methods

### Data Collection

Social network studies seeking to evaluate the global network structure of a community of people require data on the presence—or absence—of social interactions among the members of the population studied (Wasserman and Faust, 1994; Quatman and Chelladurai, 2008a). A major validity threat is researchers' determination of the actors to be included in the SNA. Researchers must identify an accurate list of actors and capture as many ties among the actors as possible for an accurate representation of the network structure (Quatman and Chelladurai, 2008b). To capture as many potential actors in the physical literacy scholarship domain as possible, we collected data through a systematic search and selection process to address our broad research purpose (akin to the identification and selection steps of a systematic scoping review; Levac et al., 2010).

Data collection occurred June 1 through July 31, 2019. We retrieved the list of articles published prior to June of 2019, with the Preferred Reporting Items for Systematic reviews and Meta-Analyses (PRISMA) used as a guide (Liberati et al., 2009). To identify articles related to physical literacy, six major online research databases were searched (i.e., PsycINFO, PsycARTICLES, Cochrane Library, PubMed/MEDLINE, SPORTDiscus, and CINAHL). Use of a predominant search term is considered an acceptable strategy for detecting relevant materials with sensitivity and precision during a systematic search and selection process (Dieste et al., 2009). In light of our explicit focus on scholars advancing the physical literacy paradigm, we employed “physical literacy” as our search term.

Criteria for inclusion in this study were: (1) published in peer-reviewed journals; (2) written in English language; and (3) utilized the phrase “physical literacy” in the title or abstract. Technical reports, book chapters, abstracts, conference presentations, and community resources were excluded. Full-text digital copies for 11 articles could not be retrieved via any available sources (e.g., interlibrary loan) and were also excluded^1^.

**Figure 1:**
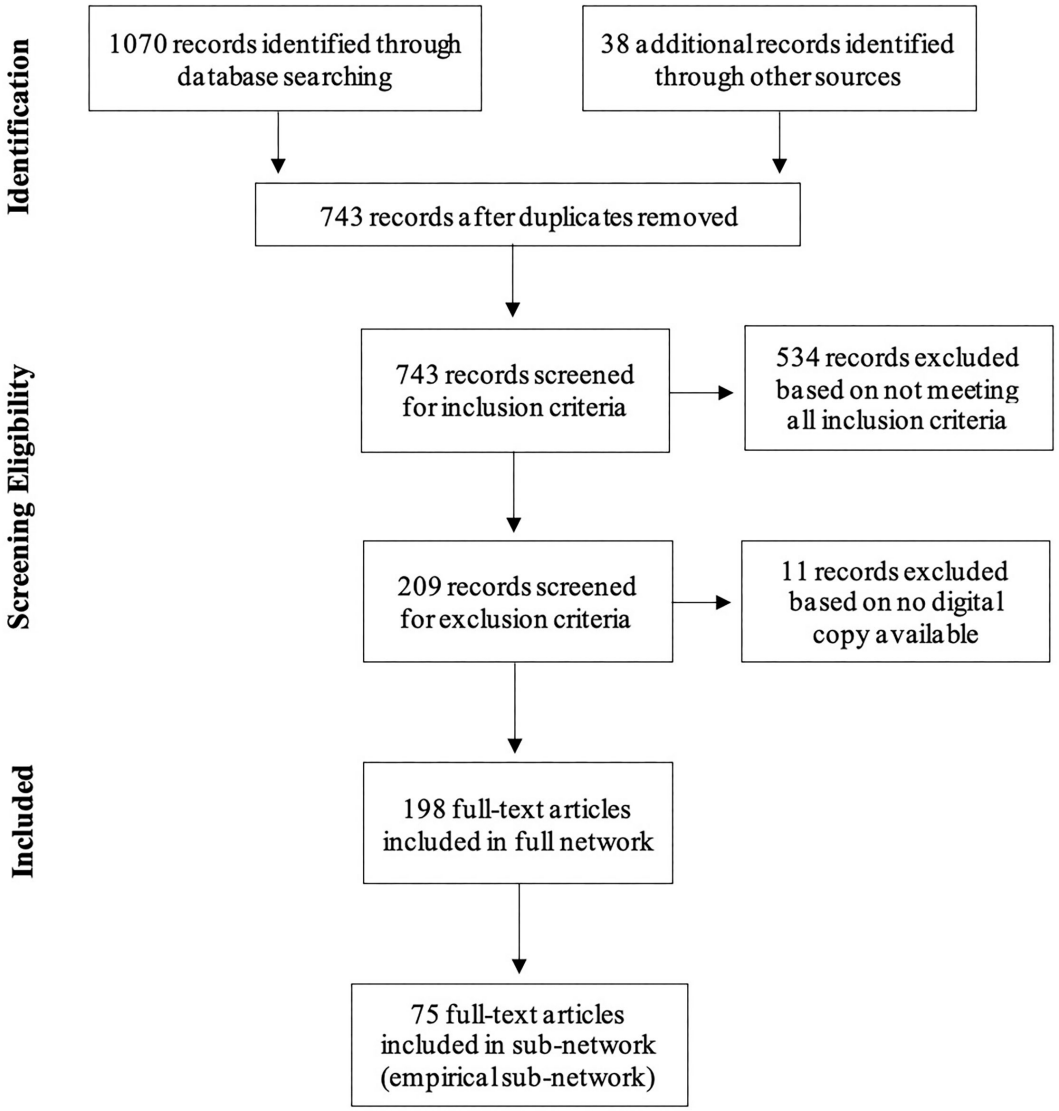
Preferred Reporting Items for Systematic Reviews and Meta-Analyses flow diagram.

Data collection resulted in a total of 1,070 records retrieved. A backward search of reference lists from the eligible records resulted in retrieving an additional 38 records. Preliminary screening identified 365 duplicate records, once removed a total of 743 records were screened for inclusion and exclusion criteria. Upon screening, 545 records were excluded and 198 articles included in the full network (see Figure 1)^2^. Among the 198 articles included in the global network, 75 articles represented empirical studies used to create the sub-network^3^.

### Social Network Analysis of Authors Shaping Physical Literacy Literature

The structure of relational webs is a central concept within global social networks (Balkundi and Harrison, 2006). The social network structure illuminates the pattern of interactions among individual actors within the social network. We operationalized the identified authors during our systematic search and selection process as actors (i.e., nodes) in the network structure. The number of times authors published with one another served as the relationship of interest (i.e., represented as ties between nodes). To construct the network, we created a full list of authors identified and converted it to a matrix which quantified all authors and the number of times each co-authored with the other authors. This global co-authorship matrix was designed to be an undirect network in that co-authorship is always reciprocal. A sub-network matrix consisting of the empirical studies was also constructed using the same methods. We explored these global social network and sub-network structures through quantification and visualization techniques using UCINET software (Borgatti et al., 2002), Pajek and Gephi to evaluate macro- and component-level network properties (Chen and Jackson, 2018). See Table 1 for descriptions of these network properties.

**Table 1:** Definitions of network measurement.

**Macro-level**	
Density	The ratio of the number of connections within or between groups to the total number of connections possible; this ratio is often described as the likelihood of collaboration for two given authors indicating a larger density network has a higher probability that two given scholars collaborate (Chen and Jackson, 2018).
Clustering coefficient	The mean of the clustering coefficient of all the actors; this mean is evaluated by three times the number of triangles in the co-authorship network divided by the number of connected sub-graph consisting of three actors and two or three lines; It indicates how likely it is that a co-author of a certain author would also be a co-author of another one's co-author (Chen and Jackson, 2018).
Average degree	The ratio of twice the number of connections to the number of actors on average; the expected number of coauthors that any scholar in this network will have implying the overall degree of network collaboration (Chen and Jackson, 2018).
**Component-level**	
# of components	A subset of the network that all nodes are linked directly or indirectly.
Newman's modularity	Modularity evaluates the strength of a subnetwork division; co-authorship networks with high modularity have subnetworks with authors that are more likely to collaborate together whereas scholars in low modularity networks are less likely to work together (Chen and Jackson, 2018). There are various ways to assess modularity and, in this study, Newman's modularity was used to calculate the strength of subgroups of co-authorship network in physical literacy. Mathematically, modularity is calculated as the sum of the difference between the actual number of links between two nodes and the expected number of edges for all node pairs as illustrated in equation. See Newman (2006) for further information about this algorithm.

For the analysis, we explored general collaboration trends by investigating the number of articles, authors, co-authored authors, isolates, frequency of co-authored pairs, and average number of authors per publication through network matrices and visualizations (Quatman and Chelladurai, 2008b). Second, we measured different network properties to explore structural patterns at multi-levels. At the macro-level, we examined density, overall clustering coefficient, average degree, and average path length to grasp properties of the whole network (Quatman and Chelladurai, 2008b; Love and Andrew, 2012; Chen and Jackson, 2018). At the component-level, we measured number and ratio of components, and Newman's modularity using Gephi, component separation (Quatman and Chelladurai, 2008b; Love and Andrew, 2012; Chen and Jackson, 2018). Even though the concept of components is related to clusters, they should be differentiated. A component presents a group of authors that are connected through co-authorship including more indirect connections, whereas a cluster represents a group of authors mutually engaged in common research (Chen and Jackson, 2018).

## Results

General collaboration trends of the full network indicate 408 authors in 198 selected articles with 2,892 ties, whereas a total of 29 isolates were identified as a single author. The average number of authors per publication was 2.06. A total of 784 pairs of authors coauthored at least twice, 390 pairs three times, 314 pairs four times, 240 pairs five times, 126 pairs six times, 24 pairs seven times, 18 pairs eight times, six pairs 10 times, four pairs 14 times, and two pairs collaborated at least 17 times, which was the maximum frequency of co-authorship.

Within the empirical sub-network, we identified a total of 257 unique authors with 2,412 ties. Among them, eight authors were identified as a single author (i.e., isolate) in this sub-network. The average number of authors per publication was 3.43. A total of 490 pairs of authors collaborated at least twice, 352 pairs three times, 296 pairs four times, 228 pairs five times, 120 pairs six times, 18 pairs seven times, 16 pairs eight times, six pairs 12 times, four pairs 13 times, and two pairs at least 14 times which was the maximum frequency of co-authorship.

### Overall Network Structure, Visualizations, and Characteristics

**Figure 2:**
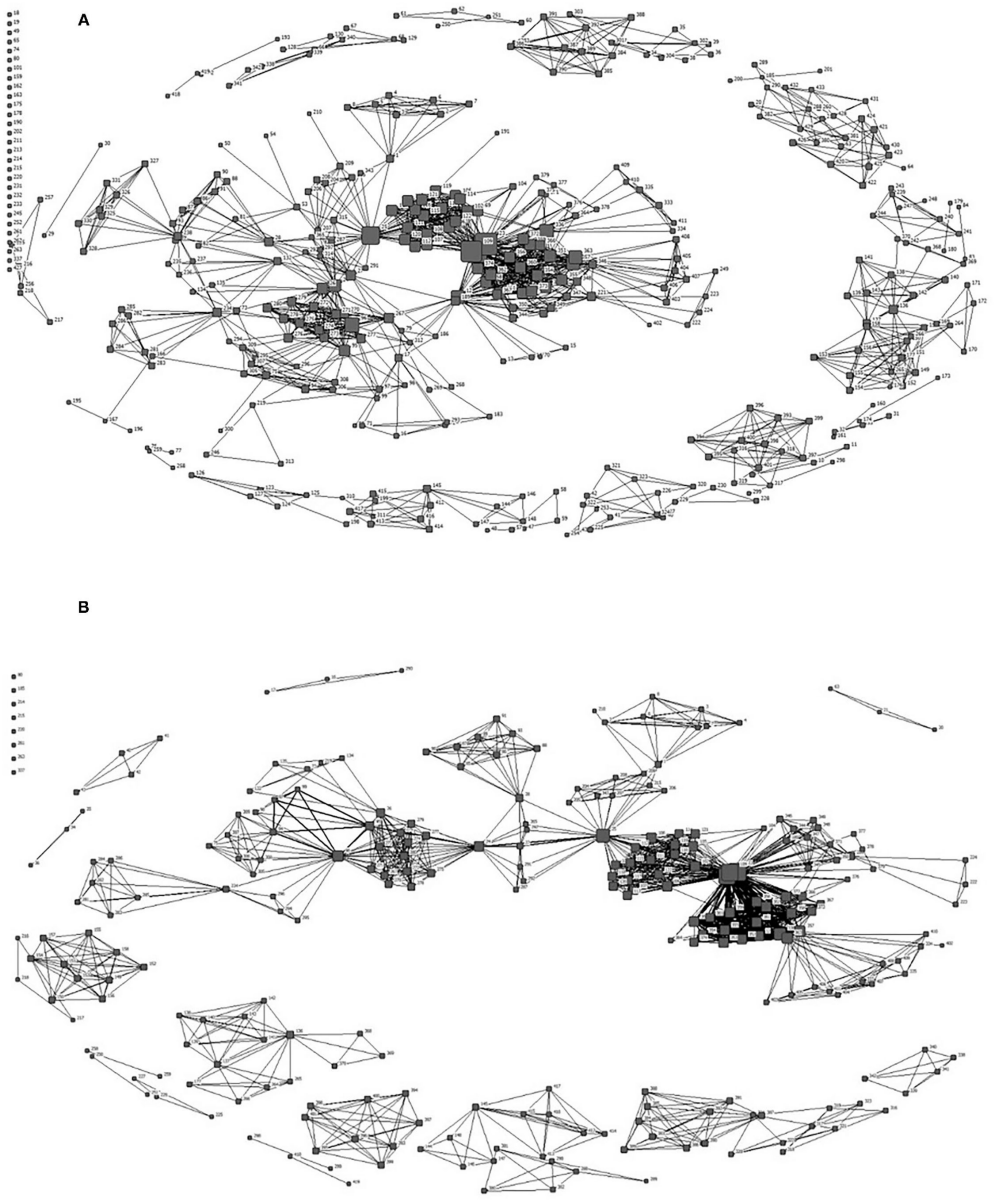
**(A)** The global co-authorship network of the full network. **(B)** The co-authorship network of the full network with only the nodes (actors) coauthored more than twice.

**Figure 3:**
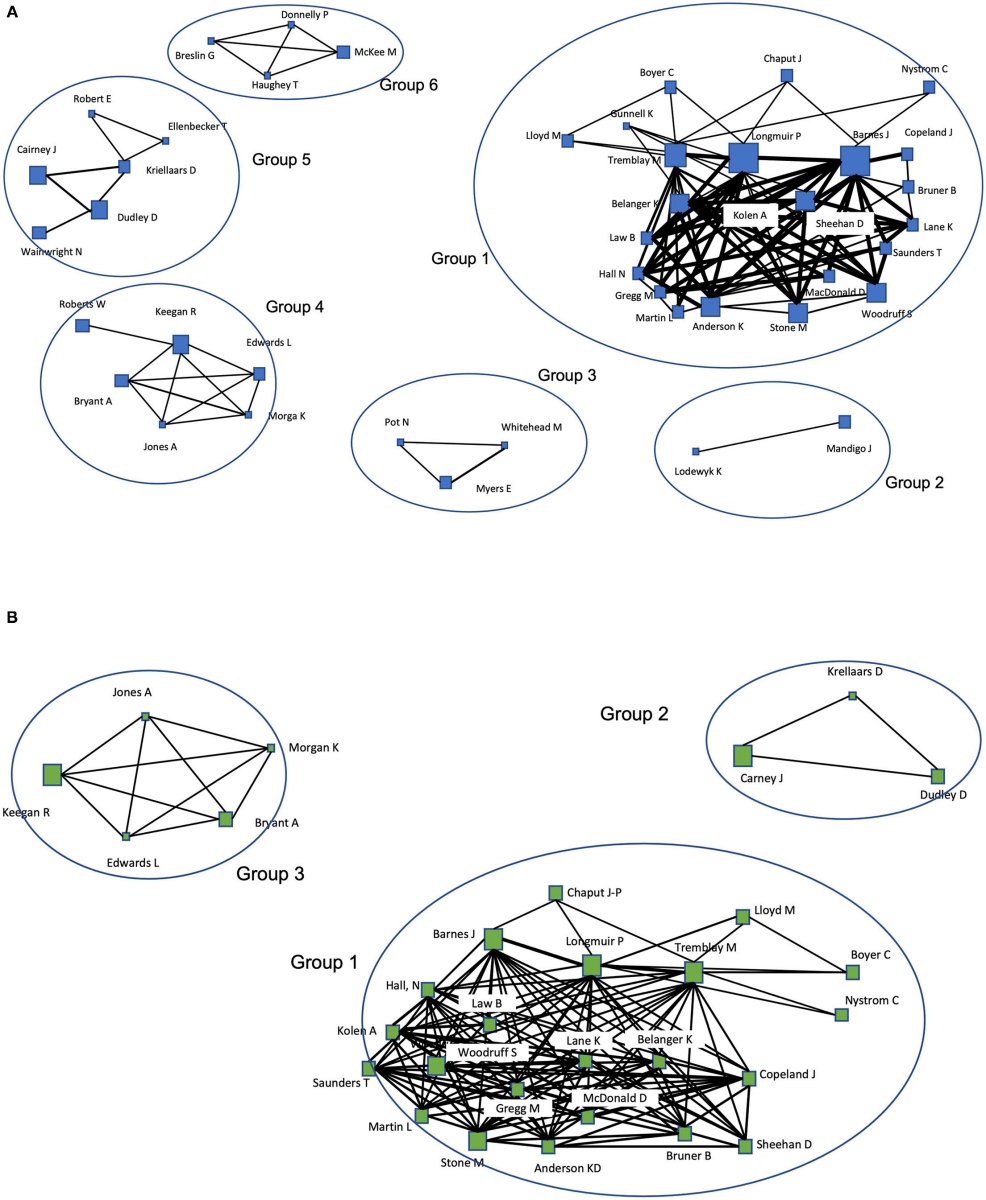
**(A)** The co-authorship network of the empirical sub-network. **(B)** The co-authorship network of the empirical sub-network with only the nodes (actors) coauthored more than twice.

Figures 2, 3 present the global co-authorship network of the full network and empirical sub-network, respectively. In these figures, node sizes reflect their degrees (i.e., number of connections) and edge widths reflect the tie strength (i.e., frequency of co-authorships) between two scholars. As presented in Figure 2A, the full network included too many nodes and ties to investigate the patterns of general collaboration trends. So, we used multiple layout techniques based on the tie strength (see Figure 2B) to investigate groups of authors that collaborate more frequently. Unlike co-authorship networks in areas such as regional science (Chen and Jackson, 2018) or sport communication (Hambrick, 2017) that are composed of very few giant communities, the full network of co-authorship in physical literacy consisted of several relatively smaller components. These findings suggest the scholarly network of physical literacy is still relatively fragmented with opportunity to build further connections across collaborations.

As indicated in Figure 2B, most components tended to be composed of authors from mostly the same country (i.e., UK, Canada) or same continent (i.e., Europe) whereas one component (Group 5) included authors from more diverse areas. Group 1 (Canada), Group 2 (Canada), Group 3 (UK and one from Netherlands), Group 4 (UK, and one from Australia), and Group 6 (UK and Ireland) included authors from mostly homogeneous countries. On the other hand, Group 5 consisted of authors from diverse geographical areas including two from the US, two from Canada, one from Australia, and one from the UK. Regarding tie strength, we note several pairs coming from the UK. All of these authors are affiliated with colleges of sport/exercise sciences or physical education at institutions in the UK.

As presented in Figure 3A, the empirical sub-network was much smaller than the full network. Figure 3B shows the co-authorship network with only scholars co-authored more than twice. The composition of Group 1 was almost identical to Group 1 within the full network, with the exception of one author from Australia. Publications from Group 1 were primarily found in the BMC Public Health special issue on physical literacy, with physical literacy defined through Canada's physical literacy consensus statement (reflective of a Whiteheadian conceptualization). Research from Group 1 demonstrated a focus on health promotion through keywords such as sedentary behavior, body mass index, childhood obesity, population health intervention, and health promotion. Members of Group 1 were responsible for the development of the Canadian Assessment of Physical Literacy (CAPL) through their work in the Healthy Active Living and Obesity Research Group.

Group 2 was composed of two researchers from Canada and one from Australia (associated with Group 5 in the full network). To frame the physical literacy construct, these authors synthesized the many definitions of physical literacy found in the literature. Research from Group 2 reflected an integration of health and education, with keywords including physical activity promotion, free play, sport, and youth development. Similar to the measurement contribution of Group 1, members of Group 2 were responsible for the development of the Physical Literacy Assessment for Youth (PLAY) tools. Members of Group 2 were also part of the Physical Literacy Research Group, responsible for the special issue on physical literacy in the Journal of Teaching in Physical Education.

Group 3 included three researchers from the UK, one from Canada, and one from Australia (associated with Group 4 in the full network). Research from Group 3 seemed to focus on clarifying the definitions, foundations, and measures of physical literacy to inform policy and practice. This was illustrated by the authors' engagement with prominent groups working to promote and develop physical literacy, contribution of multiple systematic reviews of physical literacy literature, and use of keywords such as definition, policy, practice, and international. Further, members of Group 2 developed a new definition of physical literacy sensitive and appropriate to the Australian context, that can also contribute to productive scholarly debate and conceptual development of the physical literacy construct.

### Network Properties at the Macro-Level

The density of the network structure is based upon the degree of interconnectedness among all possible social ties within the social network (Scott, 2000). The density of the full network is 0.022, which was relatively low, implying many potential collaboration opportunities are available within the full network. Yet, the generalization should be made cautiously as there are no other networks for comparison. A given scholar had roughly seven coauthors based on the average degree of 7.088. The empirical sub-network had a relatively high density of 0.044. Additionally, each author had approximately nine coauthors in that the average degree of the sub-network was 9.385.

Considering the “six degrees of separation” (Barabasi, 2002), the co-authorship network in physical literacy tended to be a small world. This suggests that subgroups of both the full network and empirical sub-network were relatively well-connected and it is more likely authors in those groups collaborate with each other. High values of the clustering coefficient of both networks (1.570, 1.560) also reflected that co-authors of a co-author were more likely to publish together often. However, the cohesiveness of the overall network was limited due to the high number of subgroups that were not directly connected to each other (see Figure 2B).

### Network Properties at the Component-Level

A component of a network is a subset of the network in which all actors are linked directly or indirectly. The full co-authorship network was not a completely connected network. There were 77 components (excluding isolates) and 28 components in the empirical sub-network (excluding isolates). For Newman's modularity, a relatively high value within a co-authorship network component indicates a network consists of authors that are more likely to publish papers together while a low value indicates authors are less likely to work together. The results show that scholars within the physical literacy co-authorship network were more likely to collaborate, having relatively high values of Newman's modularity for the full network (0.695) and empirical sub-network (0.630).

## Discussion

Through SNA of the physical literacy scientific community, the network structure can reveal existing research silos and potential unifying pipelines to prompt cooperative efforts around a scientific paradigm necessary for physical literacy to be systematically leveraged for global application (Crane, 1988; Quatman and Chelladurai, 2008a; Johnson et al., 2016). To advance these aims, we conducted co-authorship network analysis to measure the underlying social network structure of the community of physical literacy scholars, with particular focus on scholars engaged in empirical research. The physical literacy network was found highly collaborative, but still fragmented, consisting of many sub-groups of collaborators. These findings build upon Young et al.'s (2021) study of the most published physical literacy authors by capturing all authors within the physical literacy social network and examining scholars engaged in empirical research.

While Young et al. (2021) identified four highly connected clusters (human actors linked via co-authorship), initial visual observations of our full network and empirical sub-network suggest a structure comprised of several social islands (i.e., small clusters of actors connected to each other but disconnected from other clusters) and a number of social isolates (i.e., single authors disconnected from the network). When comparing Young et al.'s (2021) clusters and the groups that emerged from our co-authorship network analysis, a cluster of authors in Young et al.'s (2021) findings (e.g., Wainwright, Whitehead, Durden-Myers) was reflected in our full network (Group 3) but not our empirical network, illustrating a prominent group of actors advancing physical literacy through non-empirical scholarship. Another difference was the presence of Group 2 and Group 6 in our full network, who are contributing to the scholarly discourse and development of the physical literacy construct though not recognized by their number of publications in Young et al. (2021) or empirical scholarship in this study. As a whole, the findings reveal there is no single group of actors influencing the cohesion of the physical literacy network, rather several groups, confirming previous notions of independent research groups (Edwards et al., 2017). The multi-disciplinary nature of physical literacy and diverse contexts to which it can be applied may account for the presence of diverse collaborative groups rather than a single dominant group.

As a whole, the co-authorship network tended to be geographically bound (Quatman and Chelladurai, 2008b), with most authors coming from Canada and the UK. Particularly, Group 1 and 2 within the full network show researchers in Canada most actively collaborated with each other, while Groups 3, 4, and 5 revealed researchers in the UK engaged in collaborations outside their borders. Both the full network and empirical sub-network density were relatively low, suggesting more opportunity to collaborate and improve the underlying social network structure. Yet, the higher average degree of the empirical sub-network indicates better connection among the co-authors than the full network, which can support future collaborative efforts. Joining a research group dedicated to the advancement of physical literacy as a scientific paradigm, such as the Healthy Active Living and Obesity Research Group (associated with Group 1 in the empirical sub-network) or the Physical Literacy Research Group (associated with Group 2 in the empirical sub-network) may be an avenue to facilitate these collaborative efforts.

Within the empirical sub-network, a considerable number of researchers associated with a hospital in Canada (e.g., Tremblay, Longmuir, Barnes, Chaput, Boyer, Lloyd) and researchers from universities across Canada (e.g., Belanger, Kolen, Sheehan, Copeland)—Group 1—were identified as the most co-authored scholars (i.e., having the greatest number of collaborators) and most influential in disseminating information through the network. This finding suggests this particular collaboration may be uniquely positioned to serve as a scientific bridge within the physical literacy scholarly community. Within this cluster, Tremblay and Longmuir—the lead developers of the CAPL—were identified as the most well-connected scholars. However, the comparable closeness centrality within the full network and empirical sub-network suggests no singular leading actor in the physical literacy idea space, confirming the diversity of perspectives present.

Of the 198 articles in the full network, <40% were classified as empirical studies. These results suggest a research-practice gap, aligning with Giblin et al.'s (2014) findings of a lack of empirical evidence informing physical literacy measurement and practice. Prior to 2018, only 28 empirical studies had been published, with nine in the ICSSPE Bulletin's special issue. In 2017, a systematic review by Edwards et al. from Group 3 in the empirical sub-network concluded the current literature contains different representations of the physical literacy construct, which was consistent throughout these 28 empirical articles.

Since 2018, 47 empirical studies have been published—of which 14 came from BMC Public Health's special issue on the CAPL, contributing to Group 1's influence within the empirical sub-network. Further, seven studies came from the Journal of Teaching in Physical Education's special issue on physical literacy, contributing to Group 2's influence within the empirical sub-network. These findings demonstrate increasing interest in physical literacy evidence and intervention in the past several years. Physical literacy special issues in academic journals may foster exponential growth of the physical literacy idea space through scholars' cooperative efforts to advance physical literacy scholarship. However, these special issues may also perpetuate bias as the literature remains constrained to dominant fields (e.g., public health, physical education), viewpoints (e.g., Whiteheadian), and measures (e.g., CAPL, PLAY tools), limiting scholars' exposure to alternative ideas necessary to re-examine and improve the physical literacy paradigm (Quatman and Chelladurai, 2008b).

Despite the surge in physical literacy scholarship, confusion around its conceptualization remains (Shearer et al., 2018; Tremblay et al., 2018). Research from Group 3 in the empirical sub-network illustrates the need to clarify the physical literacy construct, as these authors conducted multiple systematic reviews of physical literacy literature to investigate the diverse definitions, foundations, and measures of physical literacy. Most intervention- and assessment-based articles in this study were grounded in the definition proposed by Margaret Whitehead (Longmuir et al., 2015; Francis et al., 2016; Edwards et al., 2018), including scholarship from Group 1 (Tremblay et al., 2018). The “Whiteheadian” conceptualization of physical literacy encompasses “the motivation, confidence, physical competence, knowledge and understanding to value, and take responsibility for maintaining purposeful physical pursuits/activities throughout the lifecourse” (Whitehead, 2013, p. 29). However, not all of these studies adopted the holistic construct laid out by Whitehead (2010), but rather represented physical literacy through various means and interventions, such as gross motor skill development (Houser et al., 2019) or cognitive responses based on Blooms' taxonomy (Alagul et al., 2012). This reductionist approach may impact the efficacy of physical literacy interventions or reflect limitations in the feasibility of adopting Whitehead's (2010) comprehensive model in practice.

In reviewing consensus statements and frameworks from the countries represented in the empirical sub-network, there were subtle differences in the conceptualization of physical literacy. Canada's Physical Literacy Consensus Statement (2015) identifies four elements—affective, physical, cognitive, and behavioral. Meanwhile, the Australian Framework posits four slightly different domains—physical, psychological, social, and cognitive (Sport Australia, 2019). Such subtle differences highlight areas in the physical literacy research space that could benefit from more collaboration within the network to establish cohesion around central tenets of physical literacy or clarify rationale for the divergence of frameworks to promote broadened understanding and application of physical literacy.

Inconsistencies throughout the body of empirical literature were highlighted further in a second systematic review by Edwards et al. (2018) from Group 3 in the empirical sub-network, which concluded that current physical literacy research consists of incompatible methodologies in measuring physical literacy. From a philosophical perspective, there is debate over whether physical literacy is observable and therefore can be measured, in light of the existential philosophical tradition put forth by Whitehead (Cairney et al., 2019b). From a practical standpoint, despite numerous attempts to measure the construct (e.g., Longmuir et al., 2015; Francis et al., 2016; Cairney et al., 2018), scholars have concluded that physical literacy cannot be assessed in a traditional sense because the methods used thus far fail to align with the holistic nature of the physical literacy construct. Without consensus on a definition, agreement on what and how to measure the construct has not yet been established (Lynch and Soukup, 2016; Edwards et al., 2018). Rather, many researchers have attempted to reduce the components of physical literacy into separate domains to calculate an overall physical literacy level (e.g., Alagul et al., 2012; De Rossi et al., 2012).

### Recommendations for Advancing Physical Literacy Research and Practice

Much of the available research thus far has focused on the physical education, public health, or sport/exercise science sectors within the youngest generations (Roetert et al., 2018). Scholars have begun extending the construct of physical literacy to different disciplines, such as nutrition (Keske et al., 2012) and rehabilitation science (Zwolski et al., 2017), and other populations, including physical education teachers (Sum et al., 2016) and older adults (Jones et al., 2018). In light of the diverse underserved populations in need of physical literacy interventions to combat physical inactivity and obesity (Mendoza-Vasconez et al., 2016; Bopp et al., 2019), interdisciplinary collaborations are needed to address this complex issue. The philosophical foundations of physical literacy have the adaptability and scope to unite disciplines around a central problem for effective physical activity promotion (Johnson et al., 2016).

However, the current study demonstrates physical literacy scholarship is a fragmented network which might impede or slow down the advancement of physical literacy strategies to address physical inactivity and obesity (Crane, 1972; Moody, 2004). While the social network of physical literacy scholars contained isolated research teams, Group 5 in the full network and Group 3 in the empirical sub-network show promise for strategic collaborations that can transcend geographic boundaries, promote interdisciplinary scholarship and research synthesis, and unite social clusters to reduce existing silos and integrate the fragmented paradigm. Such collaborations and exchange of knowledge could represent diverse perspectives to broaden and improve the physical literacy paradigm for global application.

Aligning with Whitehead's (2010) holistic approach to physical literacy, practitioners may need to consider integrating multiple dimensions of the physical literacy framework into physical activity programs to promote healthy development of the whole person (Edwards et al., 2017; Dania et al., 2020). Strategic collaborations between practitioners engaged in physical activity programs and well-connected researchers in the physical literacy network could enhance practitioner's capacity to implement evidence-based physical literacy initiatives, inform the feasibility and efficacy of holistic physical literacy interventions, and track physical activity and health outcomes to identify areas of improvement. Since <40% of the articles included in the SNA were empirical, strategic collaborations across researchers and practitioners will serve to reduce the research-practice gap and provide empirical evidence necessary to progress knowledge generation, dispersion, and application of physical literacy for effective physical activity promotion.

An approach to building connections within the fragmented physical literacy network could be engaging in productive discourse and coordinating efforts through professional associations, conferences, workshops, meetings, as well as academic and practitioner journals. The study identified several professional avenues—such as the International Physical Literacy Association—that provide an opportunity for scholars and practitioners across disciplines to collaboratively advance physical literacy. Through innovative collaboration, the physical literacy scientific community can facilitate continued growth and application of a cumulative body of knowledge to mitigate physical inactivity among underserved populations through the promotion of physical literacy.

### Study Limitations and Future Research

While the current study extends our understanding of the physical literacy idea space, the results should be interpreted with several limitations in mind. Physical literacy literature retrieved was limited to the English language due to the authors' language barriers, which may result in bias toward the Anglo-Saxon research lens. Future scholars should consider inclusion of literature written in other languages and translated variations of the term *physical literacy* for a more complete picture of physical literacy scholarship. The systematic search and selection process was also limited to six major online research databases. Broadening the search to multidisciplinary databases, such as SCOPUS or Web of Science, might capture additional literature in other disciplines. Further, the current study's focus on empirical scholarship does not account for all actors engaged in the physical literacy idea space. Future research should consider examining the social network of physical literacy scholars engaged in non-empirical work that contributes to the advancement of the paradigm.

The authors only used co-authorship to capture the individual and collaborative work of the physical literacy scientific community (Hazlett et al., 2005). Researchers may consider applying other bibliometric analysis—such as keyword mapping or citation analysis—to triangulate the findings. Future studies might also capture additional social network content to extend this line of inquiry. Such exploration may explain the gap in scholarly growth from the 1930s to 1990s, interdisciplinary research interests within sub-groups, or scholarly works that have enhanced or inhibited the diffusion of ideas. In addition, the analysis is limited to articles published in print at the point of data collection. Recent scholarship may have changed the landscape of the physical literacy social network structure and should be considered. Further, while interdisciplinary collaborations within a scientific community can advance the growth of an emerging paradigm, the social influence process may also impose barriers to the advancement of knowledge, as scholars remain constrained to the dominant paradigms subscribed by socially powerful scholars (Quatman and Chelladurai, 2008b). A better understanding of the social influence process may illuminate new avenues for innovative collaboration to facilitate continued growth of a cumulative, unified body of knowledge.

### Conclusion

The current study examined the social network of physical literacy scholars to advance the evolution, growth, research, and application of this paradigm for physical activity promotion. Study findings revealed a highly collaborative yet fragmented physical literacy network, with opportunity for greater social interactions among the scholars working in the physical literacy idea space. Of the 198 articles included in the full network, only 75 articles were classified as empirical studies. This gap between research and practice must be addressed for the physical literacy paradigm to effectively inform physical activity interventions for the goal of promoting healthy development of the whole person. Through SNA, diverse collaborative groups were identified, with implications for cooperative efforts to build a cumulative and coherent body of knowledge that enables physical literacy to be globally operationalized and leveraged for physical activity promotion. Ultimately, the physical literacy paradigm can provide a unifying framework for public health, recreation, sport, and education agencies to effectively promote physical activity among underserved populations in greatest need.

## Data Availability Statement

The raw data supporting the conclusions of this article will be made available by the authors, without undue reservation.

## Author Contributions

SB, MP, and CZ collected the data and supported manuscript development. LL-H and AK analyzed the data and drafted the manuscript. LS and CQ-Y provided critical revisions of the manuscript. All authors made substantial contributions in drafting or revising the manuscript. All authors gave final approval of the version submitted.

## Conflict of Interest

The authors declare that the research was conducted in the absence of any commercial or financial relationships that could be construed as a potential conflict of interest.

## Publisher's Note

All claims expressed in this article are solely those of the authors and do not necessarily represent those of their affiliated organizations, or those of the publisher, the editors and the reviewers. Any product that may be evaluated in this article, or claim that may be made by its manufacturer, is not guaranteed or endorsed by the publisher.

## Footnotes

^1^Please contact the corresponding author for list of excluded articles.

^2^Please contact the corresponding author for list of articles included in the full network.

^3^Please see the Supplementary Material for list of articles included in the sub-network.
