# Supplementary material for: Investigating the Social Network Structure of Physical Literacy Scholars to Advance a Paradigm for Physical Activity Promotion
Source: Front Sports Act Living. 2022 Apr 12;4:809946. doi: 10.3389/fspor.2022.809946 (PMC9039286; doi:10.3389/fspor.2022.809946)
Supplement: Supplementary file 1 [file Data_Sheet_1.DOCX]

**Supplemental Material: Empirical Sub-Network References**

1. Alagul, O., Gursel, F., & Keske, G. (2012). Dance unit with physical literacy. *Procedia – Social and Behavioral Sciences, 47*, 1135-1140.
2. Arbour-Nicitopoulos, K.P., Boross-Harmer, A., Leo, J., Allison, A., Bremner, R., Taverna, F., et al. (2018). Igniting fitness possibilities: A case study of an inclusive community-based physical literacy program for children and youth. *Leisure/Loisir, 42*(1), 69-92.
3. Bannon, C. (2013). An investigation into teaching strategies and assessment methods to foster physical literacy. *ICSSPE Bulletin Journal of Sport Science and Physical Education, 65,* 200-204.
4. Barber, W. (2018). Inclusive and accessible physical education: Rethinking ability and disability in pre-service teacher education. *Sport, Education and Society, 23*(6), 520-532.
5. Belanger, K., Barnes, J.D., Longmuir, P.E., Anderson, K.D., Bruner, B., Copeland, J.L., et al. (2018). The relationship between physical literacy scores and adherence to Canadian physical activity and sedentary behaviour guidelines. *BMC Public Health, 18*(Suppl 2), 113-121.
6. Bélanger, M., Humbert, L., Vatanparast, H., Ward, S., Muhajarine, N., Chow, A.F., et al. (2016). A multilevel intervention to increase physical activity and improve healthy eating and physical literacy among young children (ages 3-5) attending early childcare centres: The Healthy Start-Départ Santé cluser randomised controlled trial study protocol. *BMC Public Health, 16*, 1-10.
7. Brian, A., De Meester, A., Klavina, A., Irwin, J.M., Taunton, S., Pennell, A., et al. (2019). Exploring children/adolescents with visual impairments’ physical literacy: A preliminary investigation of autonomous motivation*. Journal of Teaching in Physical Education, 38*, 155-161.
8. Buckler, E.J., & Bredin, S.S.D. (2018). Examining the knowledge base and level of confidence of early childhood educators in physical literacy and its application to practice. *Early Years*. https://doi.org/10.1080/09575146.2018.1514488
9. Cairney, J., Bulten, R., King-Dowling, S., & Arbour-Nicitopoulos, K. (2018). A longitudinal study of the effect of organized physical activity on free active play. *Medicine & Science in Sports & Exercise, 50*(9), 1772-1779.
10. Cairney, J., Clark, H., Dudley, D., & Kriellaars, D. (2019). Physical literacy in children and youth – A construct validation study. *Journal of Teaching in Physical Education, 38*, 84-90.
11. Cairney, J., Clark, H.J., James, M.E., Mitchell, D., Dudley, D.A., & Kriellaars, D. (2018). The Preschool Physical Literacy Assessment tool: Testing a new physical literacy tool for the early years. *Frontiers in Pediatrics, 6*, 1-9.
12. Cairney, J., Veldhuizen, S., Graham, J.D., Rodrigues, C., Bedard, C., Bremer, E., et al. (2018). A construct validation study of PLAYfun. *Medicine & Science in Sports & Exercise, 50*(4), 855-862.
13. Caput-Jogunica, R., Lončarić, D., & De Privitellio, S. (2009). Extracurricular sports activities in preschool children: Impact on motor achievement and physical literacy. *Hrvatski Športskomedicinski Vjesnik, 24*, 82-87.
14. Choi, S.M., Sum, R.K.W., Leung, E.F.L., & Ng, R.S.K. (2018). Relationship between perceived physical literacy and physical activity levels among Hong Kong adolescents. *PloS ONE, 13*(8), e0203105.
15. Coates, J. (2011). Physically fit or physically literate? How children with special educational needs understand physical education. *European Physical Education Review, 17*(2), 167-181.
16. Conlin, G. (2013). Teacher pupil relationships. *ICSSPE Bulletin Journal of Sport Science and Physical Education, 65*, 379-384.
17. Coutinho, D., Santos, S., Goncalves, B., Travassos, B., Wong, D.P., Schöllhorn, W., et al. (2018). The effects of an enrichment training program for youth football attackers. *PloS ONE, 13*(6), e0199008.
18. Coyne, P., Dubé, P., Santarossa, S., & Woodruff, S.J. (2018). The relationship between physical literacy and moderate to vigorous physical activity among children 8-12 years. *Physical & Health Education Journal, 84*(4), 1-7.
19. Coyne, P., Vandenborn, E., Santarossa, S., Milne, M.M., Milne, K.J., & Woodruff, S.J. (2019). Physical literacy improves with the Run Jump Throw Wheel program among students in grades 4-6 in southwestern Ontario. *Applied Physiology, Nutrition, and Metabolism, 44*, 645-649.
20. De Rossi, P., Matthews, N., Maclean, M., & Smith, H. (2012). Building a repertoire: Exploring the role of active play in improving physical literacy in children. *Revista Universitaria de la Educación Física y el Deporte,* *5*(5), 38-45.
21. Dutil, C., Tremblay, M.S., Longmuir, P.E., Barnes, J.D., Belanger, K., & Chaput, J. (2018). Influence of the relative age effect on children’s scores obtained from the Canadian assessment of physical literacy. *BMC Public Health, 18*(Suppl 2), 87-98.
22. Edwards, L.C., Bryant, A.S., Keegan, R.J., Morgan, K., Cooper, S., & Jones, A.M. (2018). ‘Measuring’ physical literacy and related constructs: A systematic review of empirical findings. *Sports Medicine, 28*, 659-682.
23. Edwards, L.C., Bryant, A.S., Keegan, R.J., Morgan, K., & Jones, A.M. (2017). Definitions, foundations and associations of physical literacy: A systematic review. *Sports Medicine, 47*, 113-126.
24. Edwards, L.C., Bryant, A.S., Morgan, K., Cooper, S., Jones, A.M., & Keegan, R.J. (2019). A professional development program to enhance primary school teachers’ knowledge and operationalization of physical literacy. *Journal of Teaching in Physical Education, 38*, 126-135.
25. Francis, C.E., Longmuir, P.E., Boyer, C., Andersen, L.B., Barnes, J.D., Boiarskaia, E., et al. (2016). The Canadian assessment of physical literacy: Development of a model of children’s capacity for a healthy, active lifestyle through a Delphi process. *Journal of Physical Activity and Health, 13*, 214-222.
26. Gu, X., Chen, S., & Zhang, X. (2019). Physical literacy at the Start Line: Young children’s motor competence, fitness, physical activity, and fitness knowledge. *Journal of Teaching in Physical Education, 38*, 146-154.
27. Gu, X., Zhang, T., Cu, T.L., Zhang, X., & Thomas, K.T. (2019). Do physically literate adolescents have better academic performance? *Perceptual and Motor Skills, 126*(4), 585-602.
28. Gunnell, K.E., Longmuir, P.E., Barnes, J.D., Belanger, K., & Tremblay, M.S. (2018). Refining the Canadian assessment of physical literacy based on theory and factor analyses*. BMC Public Health, 18*(Suppl 2), 131-145.
29. Gunnell, K.E., Longmuir, P.E., Woodruff, S.J., Barnes, J.D., Belanger, K., & Tremblay, M.S. (2018). Revising the motivation and confidence domain of the Canadian assessment of physical literacy. *BMC Public Health, 18*(Suppl 2), 147-158.
30. Holler, P., Juanig, J., Amort, F., Tuttner, S., Hofer-Fischanger, K., Wallner, D., et al. (2019). Holistic physical exercise training improves physical literacy among physically inactive adults: A pilot intervention study. *BMC Public Health, 19*, 1-14.
31. Houser, N.E., Cawley, J., Kolen, A.M., Rainham, D., Rehman, L., Turner, J., et al. (2019). A loose parts randomized controlled trial to promote active outdoor play in preschool-aged children: Physical Literacy in the Early Years (PLEY) project. *Methods and Protocols, 2*(27), 1-14.
32. Hyndman, B., & Pill, S. (2018). What’s in a concept? A Leximancer text mining analysis of physical literacy across the international literature. *European Physical Education Review, 24*(3), 292-313.
33. Jones, G.R., Stathokostas, L., Young, B.W., Wister, A.V., Chau, S., Clark, P., et al. (2018). Development of a physical literacy model for older adults – A consensus process by the collaborative working group on physical literacy for older Canadians. *BMC Geriatrics, 18*(13), 1-16.
34. Keegan, R.J., Barnett, L.M., Dudley, D.A., Telford, R.D., Lubans, D.R., Bryant, A.S., et al. (2019). Defining physical literacy for application in Australia: A modified Delphi method. *Journal of Teaching in Physical Education, 38*, 105-118.
35. Keske, G., Gursel, F., & Alagul, O. (2012). Can you gain a healthy nutrition habit by physical literacy? *Procedia – Social and Behavioral Sciences, 47*, 1097-1102.
36. Kriellaars, D.J., Cairney, J., Bortoleto, M.A.C., Kiez, T.K.M., Dudley, D., & Aubertin, P. (2019). The impact of circus arts instruction in physical education on the physical literacy of children in grades 4 and 5. *Journal of Teaching in Physical Education, 38*, 162-170.
37. Lang, J.J., Chaput, J., Longmuir, P.E., Barnes, J.D., Belanger, K., Tomkinson, G.R., et al. (2018). Cardiorespiratory fitness is associated with physical literacy in a large sample of Canadian children aged 8 to 12 years. *BMC Public Health, 18*(Suppl 2), 99-111.
38. Law, B., Bruner, B., Scharoun Benson, S.M., Anderson, K., Gregg, M., Hall, N., et al. (2018). Associations between teacher training and measures of physical literacy among Canadian 8- to 12-year old students. *BMC Public Health, 18*(Suppl 2), 75-85.
39. Lloyd, R.J. (2016). Becoming physically literate for life: Embracing the functions, forms, feelings and flows of alternative and mainstream physical activity. *Journal of Teaching in Physical Education, 35*, 107-116.
40. Lodewyk, K.R., & Mandigo, J.L. (2017). Early validation evidence of Canadian practitioner-based assessment of physical literacy in physical education: Passport for Life. *The Physical Educator, 74*, 441-475.
41. Longmuir, P.E., Boyer, C., Lloyd, M., Yang, Y., Boiarskaia, E., Zhu, W., et al. (2015). The Canadian assessment of physical literacy: Methods for children in grades 4 to 6 (8 to 12 years). *BMC Public Health, 15*, 1-11.
42. Longmuir, P.E., Woodruff, S.J., Boyer, C., Lloyd, M., & Tremblay, M.S. (2018). Physical literacy knowledge questionnaire: Feasibility, validity, and reliability for Canadian children aged 8 to 12 years. *BMC Public Health, 18*(Suppl 2), 19-29.
43. Lundvall, S. (2015). Physical literacy in the field of physical education – A challenge and a possibility. *Journal of Sport and Health Science, 4*, 113-118.
44. Lynch, T., & Soukup, G.J. (2016). “Physical education”, “health and physical education”, “physical literacy” and “health literacy”: Global nomenclature confusion. *Cogent Education, 3*, 1-22.
45. MacDonald, D.J., Saunders, T.J., Longmuir, P.E., Barnes, J.D., Belanger, K., Bruner, B., et al. (2018). A cross-sectional study exploring the relationship between age, gender, and physical measures with adequacy in and predilection for physical activity. *BMC Public Health, 18*(Suppl 2), 67-74.
46. Mandigo, J., Lodewyk, K., & Tredway, J. (2019). Examining the impact of a teaching games for understanding approach on the development of physical literacy using the Passport for Life assessment tool. *Journal of Teaching in Physical Education, 38*, 136-145.
47. Mateus, N., Santos, S., Vaz, L., Gomes, I., & Leite, N. (2015). The effect of a physical literacy and differential learning program in motor, technical and tactical basketball skills. *Revista de Psicologia del Deporte, 24*(Suppl 1), 73-76.
48. McKee, M., Breslin, G., Haughey, T.J., & Donelly, P. (2013). Research into assessing physical literacy in Northern Ireland. *ICSSPE Bulletin Journal of Sport Science and Physical Education, 65*, 284-289.
49. Miller, M.B., Jimenez-Garcia, J.A., Hong, C.K., & DeMont, R.G. (2018). Process-based assessment of physical literacy and the connection to injury prevention programs. *Athletic Training & Sports Health Care, 10*(6), 277-284.
50. Millington, B. (2015). Exergaming in retirement centres and the integration of media and physical literacies. *Journal of Aging Studies, 35*, 160-168.
51. Morgan, K., Bryant, A.S., & Diffey, F.R. (2013). The effects of a collaborative mastery intervention programme on physical literacy in primary PE. *ICSSPE Bulletin Journal of Sport Science and Physical Education, 65*, 141-154.
52. Myers, E. (2013). ICT and physical literacy: The use of podcasts as an educational tool to promote motivation and raise attainment in developing knowledge and understanding in physical education. *ICSSPE Bulletin Journal of Sport Science and Physical Education, 65*, 205-214.
53. Myers, E. (2013). Motivation and physical literacy: How can motivation levels of female pupils be improved within KS3 basketball physical education lessons? *ICSSPE Bulletin Journal of Sport Science and Physical Education, 65*, 183-199.
54. Li, M., Sum, R.K.W., Wallhead, T., Ha, A.S.C., Sit, C.H.P., & Li, R. (2019). Influence of perceived physical literacy on coaching efficacy and leadership behavior: A cross-sectional study. *Journal of Sports Science and Medicine, 18*, 82-90.
55. Lloyd, R.J. (2016). Becoming physically literate for life: Embracing the functions, forms, feelings and flows of alternative and mainstream physical activity. *Journal of Teaching in Physical Education, 35*, 107-116.
56. Newton, A., & Bassett, S. (2013). Embedding physical literacy in teacher education at the University of Bedfordshire. *ICSSPE Bulletin Journal of Sport Science and Physical Education, 65*, 266-271.
57. Nyström, C.D., Baarnes, J.D., & Tremblay, M.S. (2018). An exploratory analysis of missing data from the Royal Bank of Canada (RBC) Learn to Play – Canadian Assessment of Physical Literacy (CAPL) project. *BMC Public Health, 18*(Suppl 2), 159-167.
58. Nyström, C.D., Traversy, G., Barnes, J.D., Chaput, J., Longmuir, P.E., & Tremblay, M.S. (2018). Association between domains of physical literacy by weight status in 8- to 12-year-old Canadian children. *BMC Public Health, 18*(Suppl 2), 123-130.
59. Pohl, D., Alpous, A., Hamer, S., & Longmuir, P.E. (2019). Higher screen time, lower muscular endurance, and decreased agility limit the physical literacy of children with epilepsy. *Epilepsy & Behavior, 90*, 260-265.
60. Robinson, D.B., Randall, L., & Barrett, J. (2018). Physical literacy (mis)understandings: What do leading physical education teachers know about physical literacy? *Journal of Teaching in Physical Education, 37*, 288-298.
61. Root, H.J., Valovich McLeod, T.C., Beltz, E., Murland, J., & DiStefano, L.J. (2018). The relationship between physical literacy measures of balance and the balance error scoring system in youth sports participants. *Athletic Training & Sports Health Care, 10*(6), 270-276.
62. Saunders, T.J., MacDonald, D.J., Copeland, J.L., Longmuir, P.E., Barnes, J.D., Belanger, K., et al. (2018). The relationship between sedentary behaviour and physical literacy in Canadian children: A cross-sectional analysis from the RBC-CAPL Learn to Play study. *BMC Public Health, 18*(Suppl 2), 45-65.
63. Shearer, C., Goss, H.R., Edwards, L.C., Keegan, R.J., Knowles, Z.R., Boddy, L.M., et al. (2018). How is physical literacy defined? A contemporary update. *Journal of Teaching in Physical Education, 37*, 237-245.
64. Stearns, J.A., Wohlers, B., McHugh, T.F., Kuzik, N., & Spence, J.C. (2019). Reliability and validity of the PLAYfun tool with children and youth in northern Canada. *Measurement in Physical Education and Exercise Science, 23*(1), 47-57.
65. Stone, M.R., Houser, N.E., Cawley, J., Kolen, A.M., Rainham, D., Rehman, L., et al. (2019). Accelerometry-measured physical activity and sedentary behaviour of preschoolers in Nova Scotia, Canada. *Applied Physiology, Nutrition, and Metabolism, 44*(9), 1005-1011.
66. Sum, R.K.W., Ha, A.S.C., Cheng, C.F., Chung, P.K., Yiu, K.T.C., Kuo, C.C., et al. (2016). Construction and validation of a perceived physical literacy instrument for physical education teachers. *PLoS ONE, 11*(5), 1-10.
67. Tonna, L. (2013). Physical literacy: The Maltese perspective. *ICSSPE Bulletin Journal of Sport Science and Physical Education, 65*, 337-343.
68. Tremblay, M.S., Costas-Bradstreet, C., Barnes, J.D., Bartlett, B., Dampier, D., Lalonde, C., et al. (2018). Canada’s physical literacy consensus statement: Process and outcomes. *BMC Public Health, 18*(Suppl 2), 1-18.
69. Tremblay, M.S., Longmuir, P.E., Barnes, J.D., Belanger, K., Anderson, K.D., Bruner, B., et al. (2018). Physical literacy levels of Canadian children aged 8-12 years: Descriptive and normative results from the RBC Learn to Play-CAPL project. *BMC Public Health, 18*(Suppl 2), 31-44.
70. Vašíčková, J. & Hřibňák, M. (2013). Physical literacy from the perspective of Czech pupils and teachers: Results from a pilot study. *ICSSPE Bulletin Journal of Sport Science and Physical Education, 65*, 321-325.
71. Wainwright, N., Goodway, J., Whitehead, M., Williams, A., & Kirk, D. (2018). Laying the foundations for physical literacy in Wales: The contribution of the Foundation Phase to the development of physical literacy. *Physical Education and Sport Pedagogy, 23*(4), 431-444.
72. Wainwright, N., Goodway, J., Whitehed, M., Williams, A., & Kirk, D. (2016). The Foundation Phase in Wales – A play-based curriculum that supports the development of physical literacy. *Education 3-13, 44*(5), 513-524.
73. Washburn, R., & Kolen, A. (2018). Children’s self-perceived and actual motor competence in relation to their peers. *Children, 5*(6), 72-80.
74. Yi, K.J., Cameron, E., Patey, M., Loucks-Atkinson, A., Loeffler, T.A., McGowan, E., et al. (2019). University-based physical literacy programming for children: Canadian community stakeholders’ recommendations. *Health Promotion International, 34*(5), 992-1001.
75. Zwolski, C., Quatman-Yates, C.C., & Paterno, M.V. (2017). Resistance training in youth: Laying the foundation for injury prevention and physical literacy. *Sports Health*, *9*(5), 436-443.
